# Groin injecting among a community-recruited sample of people who inject drugs in Thailand

**DOI:** 10.1186/1747-597X-9-4

**Published:** 2014-01-16

**Authors:** Lianping Ti, Kanna Hayashi, Karyn Kaplan, Paisan Suwannawong, Evan Wood, Thomas Kerr

**Affiliations:** 1 British Columbia Centre for Excellence in HIV/AIDS, St. Paul’s Hospital, 1081 Burrard Street, Vancouver, BC V6Z 1Y6, Canada; 2 School of Population and Public Health, University of British Columbia, 2206 East Mall, Vancouver, BC V6T 1Z3, Canada; 3 Interdisciplinary Studies Graduate Program, University of British Columbia, 6201 Cecil Green Park Road, Vancouver, BC V6T 1Z1, Canada; 4 Thai AIDS Treatment Action Group, 18/89 Vipawadee Road, Soi 40 Chatuchak, Bangkok 10900, Thailand; 5 Department of Medicine, University of British Columbia, St. Paul’s Hospital, 1081 Burrard Street, Vancouver, BC V6Z 1Y6, Canada; 6 Urban Health Research Initiative, BC Centre for Excellence in HIV/AIDS, 608 - 1081 Burrard Street, Vancouver, BC V6Z 1Y6, Canada

**Keywords:** Groin injection, People who inject drugs, Midazolam, Police, Thailand

## Abstract

**Background:**

Use of the femoral vein for the injection of illicit drugs (i.e. groin injecting) has been linked to various health-related harms, including deep vein thrombosis. However, little is known about the prevalence of groin injecting and factors that predict this practice among people who inject drugs (PWID) in Thailand. We sought to investigate the prevalence and factors associated with groin injecting in Bangkok, Thailand.

**Methods:**

Data were derived from the Mitsampan Community Research Project in Bangkok between July and October 2011. Multivariate logistic regression was used to identify factors associated with groin injecting in the last six months.

**Results:**

Among 437 participants, 34.3% reported groin injecting in the last six months. In multivariate analyses, factors positively associated with groin injecting included: having higher than secondary education (adjusted odds ratio [AOR] = 1.59; 95% confidence interval [CI]: 1.00 – 2.56), weekly midazolam injection (AOR = 8.26; 95% CI: 5.04 – 14.06), and reports of having had drugs planted on oneself by police (AOR = 2.14; 95% CI: 1.37 – 3.36).

**Conclusions:**

Over one-third of our sample of Thai PWID reported recent groin injecting. Frequent midazolam injection and higher education were found to be associated with groin injecting. That high intensity PWID were more likely to inject in the groin is concerning given the known negative consequences associated with the groin as a site of injection. Additionally, PWID who reported drug planting by police were more likely to inject in the groin, suggesting that reliance on law enforcement approaches may undermine safe injection practices in this setting. These findings highlight the need for evidence-based interventions to address the harms associated with groin injecting, including efforts to alert PWID to risks of groin injecting, the distribution of appropriate injecting equipment, and efforts to encourage use of other injecting sites.

## Background

In recent years, there has been growing concern over the use of the femoral vein for intravenous access (i.e. groin injecting) by people who inject drugs (PWID). While the groin is rarely the initial site of injection for PWID, there is often a progression towards groin injecting after years of continued injecting [1]. The most probable reason for this progression may be related to the physical health complications associated with repeated injecting in other sites, including the loss or perceived loss of peripheral vein access [1,2].

Historically, groin injection has been described as an extremely high-risk behaviour and a “last resort” for many PWID [3]; yet more recently, the use of the femoral vein as a site of injection has become increasingly normalized among PWID populations [4]. Previous research have identified reasons for the increasing use of groin injecting, which include: the groin being a reliable site of injection, and that it allows for a convenient and speedy injection especially among PWID who inject in public [4,5]. Furthermore, the groin appears to be a discreet site of injection, and allows track marks to remain hidden from the public and police [4,6].

In Thailand, there has been a rise in use of midazolam among PWID [7,8]. Midazolam is a fast, short-acting benzodiazepine that has potent amnesic and sedative properties [9]. The increasing use of midazolam is believed to be a consequence of the Thai government’s reliance on heavy drug law enforcement [7], which has indirectly affected the availability and pricing of heroin and other illegal drugs [10,11]. The low price of midazolam, and the fact that midazolam is easy to acquire as a licit drug, makes this substance an appealing alternative to heroin for Thai PWID [8]. The use of this particular drug may be a concern given that there may be health-related complications (e.g., venous blockage leading to amputation) that could adversely impact the health of PWID [12].

The normalization, and subsequently, the apparent increasing use of groin injection by PWID represents a serious public health issue. Research suggests that groin injecting is associated with a wide array of health problems, including deep vein thrombosis [13,14], leg ulcers [15], venous gangrene [16] and injection-related infections [17]. In addition, its close proximity to the femoral nerve increases the risk of nerve damage and related complications [2,16]. Despite the above issues, the practice of groin injecting has not, to our knowledge, been extensively documented among PWID in Asia, a setting where evidence of police corruption and violence have been observed [11]. For instance, studies have indicated that police in Thailand have been guilty of planting drugs on suspected PWID to extort money or provide grounds for arrest [18]. We sought to identify the prevalence and factors associated with groin injecting among a community-recruited sample of PWID in Bangkok, Thailand.

## Methods

The Mitsampan Community Research Project is a collaborative research project involving the Mitsampan Harm Reduction Center (Bangkok, Thailand), the Thai AIDS Treatment Action Group (Bangkok, Thailand), Chulalongkorn University (Bangkok, Thailand), and the British Columbia Centre for Excellence in HIV/AIDS (Vancouver, Canada). During July and October of 2011, the research partners undertook a cross-sectional study involving 440 community-recruited PWID who were recruited through peer-based outreach efforts and word-of-mouth. Individuals residing in Bangkok or adjacent provinces who had injected drug(s) in the past six months were eligible for participation in the study. All participants provided oral informed consent and completed an interviewer-administered questionnaire eliciting information about demographic characteristics, drug use, HIV risk behaviour, and criminal justice system exposure. The survey instrument was developed in consultation with peer researchers, which involved brainstorming key issues in the community and designing a questionnaire to reflect the community’s concerns. Prior to finalizing the survey instrument, follow-up discussions, piloting, and fine-tuning were conducted to ensure the accuracy and feasibility of the instrument. Additionally, language discrepancies between English and Thai versions were adjusted with the assistance of bilingual co-authors (KK, PS) of the manuscript. While this survey instrument has yet to be officially validated, our findings using the same questionnaire have been consistent with prior research conducted in Bangkok [19,20]. Upon completion of the questionnaire, participants were provided with a stipend of 350 Thai Baht (approximately $11 USD). The study has been approved by the research ethics boards at Chulalongkorn University and University of British Columbia.

For the present analysis, the outcome of interest was groin injection in the past six months. We compared PWID who had and had not injected in the groin using bivariate statistics and multivariate logistic regression. All participants who completed the survey between July and October 2011 were eligible for inclusion. Variables considered included: median age (≥ 38 years vs. < 38 years), gender (male vs. female), higher than secondary level education (≥ secondary education vs. < secondary education), heroin injection (> weekly vs. ≤ weekly vs. none), midazolam injection (> weekly vs. ≤ weekly vs. none), methamphetamine injection (> weekly vs. ≤ weekly vs. none), length of injecting career (years), binge drug use (yes vs. no), syringe sharing (yes vs. no), injecting in public places (yes vs. no), having had a non-fatal overdose (yes vs. no), reporting needing help injecting (yes vs. no), injected with others on a frequent basis (> 75% of the time vs. ≤ 75% of the time), ever experienced barriers accessing healthcare services (any vs. none), and reported a history of drug planting by police (i.e., having drugs planted on oneself by police) (yes vs. no). All variables refer to the previous six months unless otherwise indicated. All variables refer to the previous six months unless otherwise indicated. Binge drug use refers to having injected drugs more than usual. PWID who injected in public places were coded as “yes” if they injected drug(s) in the following places: public washroom, under highways, in bush/jungle, parking lot, abandoned building, bus, phone booth, shopping mall, and temple. Barriers to accessing healthcare were defined as in a previous study [21], and included barriers such as: long wait lists/times, stigma and discrimination by healthcare professionals, among others. ‘Drug planting by police’ was included as a potential explanatory variable given that anecdotal evidence from a study in Mexico, a setting with a similar law enforcement approach to drug control as Thailand, suggested an association between a history of negative experiences with law enforcement and groin injecting [6].

To examine bivariate associations between each independent categorical variable and groin injecting, we used the Pearson X^2^ test. Fisher’s exact test was used when one or more of the cells contained values less than or equal to five. For continuous variables, we used simple logistic regression. We applied an *a priori*-defined statistical protocol based on examination of the Akaike Information Criterion (AIC) and *p*-values to construct an explanatory multivariate logistic regression model. First, we constructed a full model including all variables analyzed in bivariate analyses. After noting the AIC of the model, we removed the variable with the largest *p*-value and built a reduced model. We continued this iterative process until no variables remained for inclusion. We selected the multivariate model with the lowest AIC score. Given previous work suggesting a strong association between drug planting by police and midazolam injecting [18], as a subanalysis, we examined potential interaction effects between drug planting by police and midazolam injection in the previous six months. We also ran a second multivariate logistic regression model that included a history of midazolam injection (yes vs. no) in replacement of the variable ‘weekly midazolam injection in the previous six months’ on the basis that past injection of midazolam could predict future groin injection. All *p*-values were two sided.

## Results

In total, 437 individuals completed the survey and participated in this study. Three participants were excluded from the original sample given that data for these individuals were incomplete. The sample included 86 (19.7%) females. The median age of participants was 38 years (interquartile range: 34 – 48 years). Among our study sample, 34.3% reported having injected in the groin in the past six months. As indicated in Table 1, in bivariate analyses, factors significantly and positively associated with groin injecting included: higher than secondary level education (odds ratio [OR] = 1.83; 95% confidence interval [CI]: 1.20 – 2.78), > weekly heroin injection vs. none (OR = 1.83; 95% CI: 1.09 – 3.08), > weekly midazolam injection vs. none (OR = 10.93; 95% CI: 5.59 – 21.37), binge drug use (OR = 1.90; 95% CI: 1.25 – 2.90), and reporting having drugs planted by police (OR = 2.14; 95% CI: 1.43 – 3.20). Less than weekly methamphetamine injection was negatively associated with the outcome in bivariate analysis (OR = 0.60; 95% CI: 0.37 – 0.98).

**Table 1:** Bivariate analyses of factors associated with groin injection among PWID in Bangkok, Thailand (n = 437)

	**Groin injection in the last six months n (%)**			
**Characteristic**	**Yes 150 (34.3%)**	**No 287 (65.7%)**	**Odds ratio (95% CI)**	***p***
**Median age**				
≥ 38 years	80 (53.3)	151 (52.6)	1.03 (0.69 – 1.53)	0.89
< 38 years	70 (46.7)	136 (47.4)		
**Gender**				
Male	119 (79.3)	232 (80.8)	0.91 (0.56 – 1.49)	0.71
Female	31 (20.7)	55 (19.2)		
**Education level**				
≥ Secondary education	105 (70.0)	161 (56.1)	1.83 (1.20 – 2.78)	<0.01
< Secondary education	45 (30.0)	126 (43.9)		
Heroin injection*				
> Weekly	41 (27.3)	54 (18.8)	1.83 (1.09 – 3.08)	0.02
≤ Weekly	58 (38.7)	110 (38.3)	1.27 (0.81 – 2.01)	0.30
nNone	51 (34.0)	123 (42.9)	1.00 (reference)	
Midazolam injection*				
> Weekly	127 (84.7)	113 (39.4)	10.93 (5.59 – 21.37)	<0.01
≤ Weekly	12 (8.0)	67 (23.3)	1.74 (0.73 – 4.17)	0.02
None	11 (7.3)	107 (37.3)	1.00 (reference)	
Methamphetamine injection*				
> Weekly	29 (19.3)	59 (20.6)	0.79 (0.47 – 1.33)	0.38
≤ Weekly	30 (20.0)	81 (28.2)	0.60 (0.37 – 0.98)	0.04
None	91 (60.7)	147 (51.2)	1.00 (reference)	
**Length of injecting career**				
Median value (years)	19	18	1.02 (1.00 – 1.04)	0.08
IQR (years)	15 – 27	14 – 24		
Binge drug use*				
Yes	59 (39.3)	73 (25.4)	1.90 (1.25 – 2.90)	<0.01
No	91 (60.7)	214 (74.6)		
Syringe sharing*				
Yes	33 (22.0)	43 (15.0)	1.60 (0.97 – 2.65)	0.07
No	117 (78.0)	244 (85.0)		
Inject in public places*				
Yes	45 (30.0)	74 (25.8)	1.23 (0.80 – 1.91)	0.35
No	105 (70.0)	213 (74.2)		
Non-fatal overdose*				
Yes	6 (4.0)	10 (3.5)	1.15 (0.41 – 3.24)	0.79
No	144 (96.0)	277 (96.5)		
Need help injecting*				
Yes	25 (16.7)	57 (19.9)	0.81 (0.48 – 1.35)	0.42
No	125 (83.3)	230 (80.1)		
Injected with others*				
> 75% of the time	57 (38.0)	113 (39.4)	0.94 (0.63 – 1.42)	0.78
≤ 75% of the time	93 (62.0)	174 (60.6)		
**Barriers to accessing health services**				
Any	118 (78.7)	204 (71.1)	1.50 (0.94 – 2.39)	0.09
None	32 (21.3)	83 (28.9)		
**Drug planting by police**				
Yes	84 (56.0)	107 (37.3)	2.14 (1.43 – 3.20)	<0.01
No	66 (44.0)	180 (62.7)		

PWID: people who inject drugs, CI: confidence interval; IQR: interquartile range.

*Activities in the previous six months.

The adjusted estimates of factors associated with groin injection are presented in Table 2. In Model 1, factors that remained positively and independently associated with groin injecting were weekly midazolam injection (adjusted odds ratio [AOR] = 9.67; 95% CI: 5.10 – 20.06) and having drugs planted by police (AOR = 2.09; 95% CI: 1.33 – 3.29). An analysis of potential interaction effects involving drug planting by police and a history of midazolam injection was conducted but failed to yield any statistically significant effects. Alternatively in Model 2, which included a total of 439 participants with complete data, the following variables were positively and independently associated with groin injecting: higher than secondary level education (adjusted odds ratio [AOR] = 1.62; 95% CI: 1.05 – 2.53), ever injected midazolam (AOR = 4.12; 95% CI: 2.06 – 9.16), binge drug use (AOR = 1.72; 95% CI: 1.11 – 2.69), and drug planting by police (AOR = 1.89; 95% CI: 1.25 – 2.88).

**Table 2:** Multivariate logistic regression analyses of factors associated with groin injection among PWID in Bangkok, Thailand

	***Model 1 (n = 437)***			***Model 2 (n = 439)***		
**Variable**	**AOR**	** 95% CI**	***p-value***	**AOR**	** 95% CI**	***p-value***
**Education level**						
(≥ Secondary education vs. < Secondary education)	1.55	(0.97 – 2.50)	0.07	1.62	(1.05 – 2.53)	0.03
Midazolam injection*						
(> Weekly vs. None)	9.67	(5.10 – 20.06)	<0.01	-	-	-
(≤ Weekly vs. None)	1.56	(0.64 – 3.82)	0.33			
**Ever injected midazolam**						
(Yes vs. No)	-	-	-	4.12	(2.06 – 9.16)	<0.01
Binge drug use*						
(Yes vs. No)	1.43	(0.88 – 2.30)	0.15	1.72	(1.11 – 2.69)	0.02
**Drug planting by police**						
(Yes vs. No)	2.09	(1.33 – 3.29)	<0.01	1.89	(1.25 – 2.88)	<0.01

PWID: people who inject drugs; AOR: adjusted odds ratio; CI: confidence interval.

*Activities in the previous six months.

## Discussion

In the present study, we found that just over one-third of a community-recruited sample of PWID in Bangkok reported groin injecting in the past six months. Having injected in the groin was positively associated with higher than secondary level education and frequent midazolam injection. In addition, those who reported drug planting by police were also more likely to report groin injecting.

The high prevalence of groin injecting observed in our study builds on a growing body of literature demonstrating the common use of the groin as a site of injection among PWID [17,22,23]. Studies conducted in Seattle, Washington and six locations in the United Kingdom (UK) (i.e., Manchester, Bristol, Teeside, Plymouth, Exeter, and Wigan) reported that 40% and 45% of PWID in their sample had injected in the femoral vein, respectively [4,24]. Several reasons for the increasing prevalence of groin injecting among PWID have been proposed, and these relate to the wide array of physical health problems, including injection-related infections and abscesses resulting from frequent injection in peripheral veins (i.e., cubital fossa) [1].

Findings from our first and second model revealed that midazolam injection in the past six months and a history of midazolam injection was strongly associated with groin injecting, respectively. This supports our hypothesis that previous midazolam injection may predict future groin injecting among this population, given that this drug in its soluble form is highly acidic and can be damaging to veins. Our findings are consistent with previous studies that have documented that many PWID who inject midazolam have resorted to groin injection once more convenient sites become inaccessible [7]. Given the known adverse health outcomes associated with groin injecting [14,25], public health interventions should first focus on educating PWID on the risks and harms associated with this practice in addition to improving the distribution of appropriate injecting paraphernalia, including sterile needles and syringes, as well as alcohol swabs [8]. Furthermore, a recent study conducted in the UK by Zador and colleagues (2008) revealed that groin injectors were able to successfully access and use alternative peripheral injecting sites following complications due to chronic groin injecting. In an effort to encourage those engaging in high-risk femoral injecting behaviour to utilize lower-risk peripheral sites, it may be of benefit for healthcare workers to support PWID in learning how to access peripheral veins even when these sites are believed to be no longer accessible [23]. For those PWID where peripheral vein access is not possible, other harm reduction interventions such as education on safer groin injection techniques and the provision of appropriate injection equipment, such as filters and appropriate needles, may be of value in this setting [5]. Given the high degree of stigma and discrimination associated with illicit drug use in Thailand [26,27], educational efforts and materials might be best provided through existing drug user-run drop-in centres operating in Bangkok [28].

Although we expected that PWID with higher education would avoid risky injection practices (i.e., groin injecting), we found a positive association between higher than secondary level education and a history of groin injecting in our study. One possible explanation for this finding may be that these individuals may be more likely to try to hide their injecting behaviour from family, friends, healthcare workers, or law enforcement officials for fear of further discrimination.

Of concern, PWID who reported having drugs planted on them by police were more likely to report groin injecting. While in other settings this finding would likely reflect the fact that groin injecting allows PWID to hide visible track marks from law enforcement [6], previous research conducted in Thailand suggest that increasing rates of midazolam injection may be playing a role in driving the aforementioned association. Fairbairn et al. (2009) also found that a history of midazolam use was associated with evidence planting by police among Thai PWID. It was proposed that this may be attributed in part to the drowsy appearance of PWID when under the influence of midazolam, making these individuals more vulnerable and identifiable to police [18]. As well, midazolam injectors tend to be of older age and thus, may already have a history with police [7]. However, our subanalysis focused on identifying interaction effects between drug planting by police and midazolam injection failed to yield a statistically significant result. Given the limited and inconsistent available evidence examining the relationship between policing practices, midazolam use, and reporting groin injection in this setting, future research using in-depth qualitative methods should seek to explore this further. Nevertheless, given the ineffectiveness of drug law enforcement approaches in reducing drug use in Thailand [29,30], there is a clear need for alternative policy approaches to be developed, implemented and evaluated. In the meantime, efforts should be made to ensure that policing practices do not undermine existing public health efforts, including those focused on promoting safer injecting practices [31]. Additionally, given the lack of legal grounding for harm reduction services in Thailand, efforts to plan and implement a national harm reduction policy is urgently needed.

This study is limited in several ways. The study sample was not randomly selected and therefore, it may not be possible to generalize the findings of this study to Thai PWID more broadly. Additionally, given that our eligibility criteria only included individuals who had injected drugs in the past 6 months, we were unable to explore the risk factors associated with groin injection among individuals with a history of injection drug use but who had not injected drugs in the past six months. The data obtained are also based on self-reports by PWID and may be susceptible to socially desirable reporting or recall bias. We should also note that our findings were limited by the cross-sectional design of the study, and therefore, we cannot determine a temporal relationship between exposure and outcome. Finally, given the nature of the study, there may be issues related to unmeasured confounding (e.g., homelessness, problems accessing peripheral veins, mental health issues, abuse) that we were not able to capture in this analysis.

## Conclusions

In sum, we found a high prevalence of groin injecting among Thai PWID, with those who have completed higher education, those who reported frequent midazolam injecting, and those who reported drug planting by police were more likely to engage in groin injecting. Given the various adverse effects of groin injecting among PWID, these findings highlight the need for evidence-based public health interventions that focus on educating PWID on the harms associated with injecting into the groin. As well, interventions that aim to harmonize public health and policing practices are urgently needed to minimize unsafe injecting practices.

## Competing interests

The authors declare that they have no competing interests.

## Authors’ contributions

The specific contributions of each author are as follows: LT and TK were responsible for study design; LT conducted the statistical analyses and prepared the first draft of the analyses; All authors provided critical comments on the first draft of the manuscript and approved the final version to be submitted.
